# Dr. Adams on Croup

**Published:** 1812-05

**Authors:** Joseph Adams


THE
### Medical and Physical Journal.

5 OF VOL. XXVII.] MAY, 1812. [NO. 159.

*To the Editors of the Medical and Physical Journal.*

GENTLEMEN,

HAVING received from my friend, Dr. Jackson, of Boston, a copy of Dr. Hosack's valuable paper on the Croup, it was my intention to have transmitted some remarks on that subject for the American Register; but, as you have, with much propriety, given Dr. H.'s paper a place in your Journal, I shall, with your permission, avail myself of the same, and thus avoid the present uncertainty of all transmarine communication. As my remarks can only be considered supplementary to Dr. Hosack's observations, I shall introduce them with a short abstract of the American paper.

Though it should be admitted that Dr. Cullen's account of croup is short, and perhaps indistinct, from the few opportunities he had met of observing the whole progress of the disease, yet it is evident that the new membrane was, in his days, traced to the bronchia, since he speaks of it as extending to the ramifications of the trachea. At the same time it must be admitted that Dr. Hosack's correspondents have traced this substance further, and with still greater accuracy. Dr. Bard observes, that " the lungs, throughout their whole substance, partake of the affection, insomuch that he has seen those organs rendered so dense and solid, that they exhibited in their appearance a great resemblance to the firm and solid substance of the liver." Of this passage I shall hereafter take further notice.

Dr. Hosack objects to the distinction between spasmodic and inflammatory croup, and produces very good authority in support of his doctrine. Dr. Cheyne gives a very correct description of the appearances in the thorax when death has occurred after four days' illness, tracing the membrane from the larynx through the trachea and its divisions, with purulent matter in the progress of being forced up by the frequent and laborious respiration during life; the inflammatory appearance along the inner coat of the trachea, to which the membrane is attached, extending to the bronchia, the serous fluid in the cellular substance of the lungs, &c.

Dr. Cullen remarks that the disease is sometimes the effect of cynanche maligna. This is confirmed by Dr. Bard and Dr. Ferriar. Dr. Cheyne describes it as the sequela of scarlatina, of the measles, of the secondary fever of small-pox, adding that it has supervened a common catarrhal affection, and proved the attendant of a putrid thrush. In most of these he is confirmed by Dr. Rush. Dr. Foulke found it succeed acute rheumatism, and Dr. Sayre detected it in yellow fever. From these facts Dr. Hosack divides croup into idiopathic and symptomatic.

The next inquiry is whether the disease can be communicated by contagion. The result of Dr. H.'s reasoning is, that wherever contagion has occurred, the croup has been symptomatic, and the primary disease contagious; and that in croup itself contagion has been suspected, from two persons in the same family having been affected from one common cause.

Though the disease among children rarely occurs for the first time after twelve years of age, yet instances are recorded of grown persons, and of some even in advanced life.

This short abstract must be sufficient of a paper to which your readers can so readily refer.

The first consideration is the nature of the disease; and here I cannot help agreeing with Dr. Hosack, that the admission of spasmodic croup is not only gratuitous, but may sometimes be dangerous. In justice to Dr. Cullen, we ought to observe, that he considers the disease as always arising from inflammation; and that spasm is only the effect of that inflammation. His remedies are therefore all directed to the first disease, and he expressly tells us that he has never found benefit from antispasmodics. But, in this and in many other inflammatory diseases, there is reason to fear that the very term, spasm, has too often been used to cover the indecision of a timid practitioner, and to prevent the use of the only effectual remedies, and of remedies that are useless unless early applied.

The adventitious membrane being generally admitted as the cause of that sound from which the disease derives its name, it is of importance to inquire by what means such a new substance can be formed. Michaelis* is the first person who collected all that could be met with before his time which seemed referable to the disease. It is evident that he confounds two causes and two different appearances, the one of coagulated blood or lymph from hæmorrhage, and the other of lymph extravasated from inflammation. Haller, to whom he refers, was also much confused from the same

* Dissertatis Inauguralis de Angina Polyposa seu membranacea, Argentorati. P. 38.

sources.

sources. Michaelis's theory of the disease is very much like the continental pathology. At one time he adopts Dr. Warren's opinion that the membrane is inspissated mucus, and the disease scrofulous;* at another time that it is arthritic and also venereal, because, as Astruc observed before him, *virus venereum valet multum ad coagulandos humores lymphaticos, variique generis concretiones polyposas procreandas.*

It is not easy to ascertain whether the branches of veins as described by Bertolin and Tulpius, were hollow or not. It is probable that some of them were, which might have given rise to the opinion that the whole were actually veins separated, as they both express it, so nicely from the lungs, that one might have conceived it the labor of an industrious anatomist. Tulpius† gives an account also of a piece of lung which was expectorated with blood, by a physician who had taken an antimonial vomit. Morgagni‡ has a very long paper on the subject, and, as Dr. Warren very justly remarks, the number of authorities he produces shows the disease is more common than was then suspected. It is not, however, a little remarkable that Dr. W. should tell us "Morgagni is the only one who seems to have understood the disease," when the opinions of the two are so different; the English physician considering these polypi as formed of inspissated mucus, and the Italian anatomist imputing them to coagulated blood. The first of these opinions is so ably answered by Dr. Baillie§, that I shall not detain the reader on this question; the latter is very rationally defended in the cases related and referred to by Morgagni, who afterwards describes the condensed state of the lungs, as mentioned by Dr. Hosack, and conceives it may arise from innumerable polypi formed in the extremest branches of the bronchiæ and of the vesicles communicating with them.

Though, from the various histories of membranes ejected from, or found in, the trachea, there can be no doubt that more than one disease is described, yet it is but justice to add that confusion from this source has, since Dr. Home reduced the complaint to some form, been principally among the continental writers, of whom Michaelis has preserved a rich collection. It is probable that most of the polypi, if we may so call them, that is, the substances thrown up in

* Med. Trans. vol. i. p. 307.

† Lib. 2, cap. xiv.

‡ Epist. xxi. art. 20 and sequent.

§ Morbid Anatomy, p. 94. Second edition. Art. Polypus in the Trachea.

any considerable quantity, whether in branches, or imitating the substance of the lungs, were only coagulated blood or lymph, sometimes ramifying according to the branches of the bronchia, at other times coagulated almost in the act of ejecting them from the trachea. The density of the lungs may certainly arise from the number of ramifications thus filled, and from the serum effused at the same time. But that particular consolidation which produces a resemblance to the liver, and which the ancients distinguished by the term *infarction*, is, I believe, only produced by a chronic or frequently returning inflammation, which, by the effusion of coagulated lymph into the connecting cells, produces such general adhesions as gradually to prevent the expansion of the air cells, and thus render the office of the lungs more and more difficult and laborious.

That the polypi as they are called were really coagulated blood or lymph, appears the more probable, because in most instances they have been attended with a spitting of blood in a greater or less degree. Morgagni, who I believe was the first to give these substances the name of *polypi*, tells us his patient had been subject to a spitting of blood.* Even Dr. Warren, who wishes to consider the disease as inspissated mucus, informs us that his patient sometimes expectorated blood with the polypi. Mr. Saumarez's patient expectorated mucus often mixed with blood.† Though this was not the case in the instance witnessed by Th. Bartoline, yet he informs us his patient was phthisical. Both Tulpius's cases were attended with fatal hæmorrhage. In hæmoptysis, where the blood issues slowly, and is spread along the sides of the trachea or its branches, coagulation into a membranous form is the natural consequence. "When the blood," says Mr. Hunter, "oozes out on a surface, in which act it immediately coagulates, and coagulates in that form, it may then be said to form an inorganized membrane, of which there are many; and these coagula," he adds, "more especially the thin ones, cannot easily be distinguished from original membranes.‡ It has been observed, by the same author, that hæmorrhage in all cases gives a stronger disposition in the blood to coagulate quickly, and this has been finely illustrated in some of Dr. Jones's beautiful experiments.

---

* Epist. xxi. art. 19.

† Hunter on the Blood. See plate 7. It is much to be regretted that this invaluable work has not a copious index, and a reference in the text to the plates.

‡ Ibid, p. 28.

Having thus premised all that appears necessary on the subject of hæmorrhage, I shall proceed to consider the other events under which such a membrane may be formed as appears in croup. In doing this we must refer to the process of inflammation, as it appears in different parts, or in parts differently constructed. For some time past it has been a custom to adopt the division and nomenclature of M. Bichât, in the various membranes. I shall at present only notice the first and second of his divisions. These are the *mucous* membranes and the *serous*. The first is a term almost universal among physiologists. On the second, though very properly put under one class, on account of the similarity of their office, I cannot help expressing my objection to the name and the functions, as designated by this justly celebrated writer. That they sometimes throw out serum cannot be doubted ; but is not such effusion always the effect of disease, and should a part derive its name from its diseased actions? or have we any proof that the fluid with which their healthy surface is lubricated is not a secretion? M. Bichât indeed speaks of these membranes as lubricated by a lymphatic fluid ;* but a *lymphatic fluid* should either be *fluid lymph*, or its property should be described. In my remarks, therefore, I shall prefer following Mr. Hunter, at the same time showing the similarity of their opinions; though the caution of our countryman has preserved him from the inconvenience of ill defined terms.

"I formerly," says Mr. Hunter, "divided the surfaces capable of taking on inflammation into two; the first of these was the cellular membrane in general, together with the whole circumscribed cavities; the second was all the outlets in the body, called mucous membranes; for instance, all the ducts of glands, and the alimentary canal. The first order of parts generally, if not always, takes the adhesive first in the true inflammation, and then all the three in succession; for the adhesive is immediately admitted in the cellular membrane and circumscribed cavities, to exclude, if possible, suppuration, where suppuration, and of course ulceration, would prove hurtful.

"2dly. In internal canals, where adhesions in most cases

---

* Membrane serouse, caractérisée aussi par le fluide lymphatique que les lubréfie cans cesse, et qui, séparé par exhalation, de la masse du sang, differe en cela du précédent, qui s'en échappe par voie de sécrétion. *Tr. de Membrane*, p. 6.—If this fluid is lymphatic, why are the membranes called serous; and is there any proof, because on opening a recent subject we find it volatile, that therefore it is not furnished by a secreting process?

would prove hurtful, the parts run immediately into the suppurative inflammation; the adhesive being in common excluded. Such parts are the nose, mouth, trachea, outlets of the organs of secretion, which may be called mucous membranes. The matter, under inflammation, from such is generally not called true matter, or purulent, but it often is so; however this will be according to circumstances. Since those surfaces are in general secreting surfaces, suppuration would appear to be only a change in the secretion. The matter will seem to vary from true matter towards that of the common secretion of the parts, and *vice versa;* but this does not alter the position, for it is common to matter from a sore, and even common to our ordinary secretions."*

I shall now copy Bichât's words in describing these different processes according to the nature and structure of the parts, taking the liberty to transpose the two orders, for a reason which will presently appear.

"N'y a-t-il pas un parallèle exact à établir, 1°. entre les adhérences des membranes séreuses qui résultent de l'inflammation, et celles de la réunion des plaies par première intention? Ces adhérences ne sont-elles pas, dans l'un comme dans l'autre cas, l'effet de l'inflammation à son premier période? 2°. entre l'exsudation purulente de ces membranes, et la suppuration des plaies non réunies? l'une et l'autre ne sont-elles pas l'effet du second période inflammatoire? Si l'identité de ces phénomènes est reconnue, ne dépend-elle pas de l'identité de structure des membranes séreuses et du tissu cellulaire, agent essential de la réunion et de la suppuration des plaies?†

"Pourquoi, à la suite des inflammations dont elles sont le siège, les membranes muqueuses ne contractent-elles presque jamais des adhérences, comme cela arrive si souvent sur les surfaces sérouses? Pourquoi la surface interne de l'estomac, des intestins, de la vessie enflammée, ne se colle-t-elle pas alors dans ses diverses portions, comme celle de la plèvre, de la tunique vaginale, etc.?—Pourquoi dans les inflammations des membranes muqueuses, y a-t-il un écoulement abondant du fluide qui les humecte habituellement, ce qui constitue les diverses espèces de catharres, tandis que la source du fluide qui s'exhale des membranes séreuses est communément tarie dans les cas analogues? Cette seconde question répond-elle à la première?"‡

The reader will see the propriety of the transposition in these two paragraphs, because *adhesion* is referred to in the first from the second, as they stand in M. Bichât's Treatise.

Thus far our authors travel together pretty easily; both speak of the membranes lining cavities, and the cellular

* Treatise on the Blood, &c. p. 240 and 242.
† Traité de Membrane, p. 119.
‡ Id. p. 77.

membrane membrane as subject to similar laws under inflammation; and though Bichat does not use the terms adhesive and suppurative inflammation, as the first and second stages, yet he speaks of *adherence dans le premiere periode, et suppuration* as *l'effet du second periode inflammatoire.*

In describing inflammation, in mucous membranes he does not, it is true, use the term *suppurative inflammation* as taking place without previous adhesions; but he asks, why adhesions do not take place in them? and remarks the increased discharge of their habitual secretion. It is, however, certain that not only is the quantity increased but the property altered, and, as Mr. Hunter remarks, under certain stages, acquires all the characters of genuine pus; altering like the discharge of a common sore, according to the degree of inflammation.

We have not yet, however, arrived at any thing which will enable us to account for the membrane formed on the inner surface of the trachea, in croup: nor have I seen any thing in Bichat that can direct us to it. I shall therefore continue the transcript from Mr. Hunter, beginning from the passage at which we just broke off.

"If this inflammation, continues he, which produced suppuration on those surfaces, becomes more violent, or has something of the erysipelatous disposition, we find that it moves from the suppurative to the adhesive, and throws out the coagulating lymph. I have seen this in the intestines, often on the inside of intestines that had been strangulated in a hernia. I have been able, also, to produce it on the inside of the vagina of an ass, by injecting a strong solution of corrosive sublimate. But, if of the erysipelatous kind, these surfaces will take on the adhesive action immediately or at first. This is evidently the case in what is called the ulcerous sore throat; I have seen it in the trachea, I have seen it thrown up from the lungs in branches, I have seen it in the pelves of the kidneys, ureters, bladder, and urethra."

As I conceive most of your readers must be well acquainted with Mr. Hunter's important distinctions in the various kinds of inflammation, I shall only turn their recollection to a few particulars which distinguish two species, the adhesive and the erysipelatous.* The adhesive in those parts, which are its proper seat, is marked by the effusion of coagulated lymph, which confines the action from extending by uniting one surface to another. If the inflammation continues to increase, the second process is suppuration. In mucous membranes, if inflammation pursues its usual and most favorable course, the second process commences without any

* Those who wish for a familiar illustration of these doctrines, may consult Dr. Adams's Commentaries on Mr. Hunter's Treatise.—Editors.

previous previous effusion of coagulated lymph, and consequently without adhesions. But, if the inflammation in mucous membranes exceed its customary bounds, serum will be thrown out, as we find the case in erysipelatous inflammation. If it should increase further, coagulated lymph will be thrown out, as we sometimes find the termination of erysipelatous inflammation on the external surface, in which case we have a superficial slough. Not only the violence of the inflammation, but some peculiarity of constitution, or an epidemic state of the atmosphere, or the stimulus of a morbid poison, may produce such a change in the action of a mucous membrane under inflammation. Hence it is that we find such a variety of circumstances under which this membrane is thrown out under inflammation in the trachea: such of these as can be well ascertained I shall endeavour to mark with as much brevity as so complicated a subject will admit.

The most simple cause we have seen, is inflammation excited higher than is consistent with the mere increase and alteration of the customary secretion. That this is sufficient to induce effusion and coagulation of lymph, is demonstrated by the experiment above alluded to, in the vagina of an ass. Here, however, the violence to the parts was so great and so frequently repeated, that the effused coagulum was disposed in no order, though every where adherent.* But the preceding plate in the same work "exhibits the inner surface of the ilium covered by a *layer* of coagulated lymph, thrown out by the degree of inflammation which the parts had undergone,"† similar to the membrane in the trachea under croup. When this process is completed, the violence of the inflammation subsides for a time, either from the new action which has taken place or from the completion of that action. The patient now feels a temporary relief in the general symptoms, but the difficulty of breathing is increased, and the sound peculiar to the disease is heard. The fate of the patient depends now on the quantity of coagulum, its extent, and the degree of inflammation, which the other parts of the lungs have suffered, or on the return of inflammation after it had to a certain degree subsided. If the mischief has been no greater than the constitution can survive, the next process is the separation of the coagulated lymph, which loses its life, or, in other words, becomes a slough, and is gradually separated by the process of suppuration.

In such a case we suppose no other cause for the pro-

* See Treatise on the Blood, p. 297, and plate 8.

† See plate 6 of the same work.

duction duction of the membrane, but a higher degree of inflammation than is consistent with the process of suppuration, which last process takes place as the inflammation abates. This change does not differ from what we often see in a suppurating sore, where increased inflammation, arising from intemperance, exposure to cold, or any other cause, will sometimes produce an effusion of serum with a layer of coagulum, which soon becomes a slough. But it is certain that this membrane in the trachea, or slough, as it afterwards becomes, may be produced without very high inflammation. A malignant sore throat is usually attended with sloughs, which are only effusions of coagulated lymph, as we find the case in aphthæ. The small-pox is always attended with a slough in every pustule; and, when the fauces are much affected, we constantly see circumscribed sloughs, which very soon show their figure and complexion from the thinness of the cuticle, and the color of the surface on which they are seated. When the scarlet fever produces its characteristic effects on the throat, it is always by sloughs, succeeded by ulceration. Should the same disposition spread to the larynx and thence to the trachea, the consequence would be the formation of a similar slough as far as the disease extends. This is very well remarked by Dr. Heberden, who, with his accustomed accuracy, saw the appearance of the parts just as we might expect to trace it, according to the time that the inflammation had commenced on each. The velum pendulum was putrid; the tonsils were outwardly blackish and livid within; the uvula was covered with a thick mucus, resembling a membrane, which extended to the upper part of the trachea, and in the lower part appeared like mucus.*

From these considerations it is not to be wondered if the membrane, which is considered as characteristic of croup, should appear in so many other diseases. Whenever we find slough about the fauces, extensive and superficial, this membrane may be formed as a continuation of the same action. It is not less certain that, though the disease of which we are speaking derives its name from the affection of the trachea, yet its characteristic membrane will often be first discovered in the anterior parts of the throat. Mr. Field, whose papers in the memoirs of the Medical Society, do equal credit to his science and candour, describes a case of croup, in which the membranous appearance was early discoverable in the tonsils; and relates another from un-

* Commentaries, p. 27 and 32.

questionable authority, in which the disease was preceded by sloughy ulcerations in different parts of the mouth.* Mr. Rumsey's paper contains the same remark†. Hence its appearance in many fevers, nor should it excite much surprize if it has sometimes occurred in acute rheumatism. In the early stages of this, and of many other inflammatory complaints, the throat is often inflamed, and a metastasis to that part is by no means uncommon in different periods of the same disease.

There are other causes, however, which may produce this change in the process of inflammation in a mucous membrane or a common suppurating sore. Some constitutions are particularly liable to erysipelatous inflammation, which, at certain seasons and at certain ages, may fall on these parts. At other times an epidemic constitution of the atmosphere may induce such a disease; and, when this is the case, subjects in the weakest state of health, and particularly convalescents from other diseases, will be among the first victims.

This will also make a degree of ambiguity as to the question of contagion. On this occasion I cannot help referring your readers to two very valuable papers contained in the second volume of the "Transactions of a Society for the Improvement of Medical and Chirurgical Knowledge," published by Johnson, 1800. The one contains "an Account of Croup, by Mr. Rumsey, of Chesham," and the other, "Observations on Erysipelas, by Dr. Wells." There is a great reason to suspect that in both instances the diseases might be contagious, yet this would not prove that either croup or erysipelas is generally so. There are few local diseases but may, under certain circumstances, be contagious, as is perpetually proved in camps, fleets, and hospitals. In all which places we have topical diseases as well as fever, under various forms, according to the constitution of the atmosphere and the season of the year. Dysentery gives us a fair illustration of this, though in a much higher degree. As we see it in common life even during the autumnal season, we rarely have reason to suspect that it is contagious. But, when introduced into fleets or armies, it not only proves contagious, but converts every other disease into its own form.‡ A similar effect may follow wherever

* See Memoirs of Lon. Med. Society, vol. iv. p. 142 and 158.

† See Transactions of a Society for the Improvement of Medical and Chirurgical Knowledge, vol. ii. p. 28 and 29.

‡ On this subject see Sir. J. Pringle, also Dr. Blane on Diseases of Seamen, p. 18.

the the human race are crowded, or from too close an intercourse with the sick. This offers a further illustration of what was before remarked, namely, that high health is by no means necessary to produce such an inflammation, and that convalescents will be the principal sufferers during such epidemics. Thus we find some of Mr. Field's and of Mr. Rumsey's cases were children in delicate health, or convalescent from measles. Mr. Field,* after an interval of more than four years from the time he first offered his opinions to the public, feels stronger reason to suspect that the disease is contagious, and Mr. Rumsey† appears at least unwilling to offer a contrary sentiment. Towards the close of the last-mentioned gentleman's remarks on this subject, we are informed, that "although there were between thirty or forty children in the workhouse, only one had the disease." How much is it to be wished that, if so accurate a describer should recollect the particulars of that case, he would favor us with them;—Had the subject been long an inhabitant of the workhouse? what was the general standard of health with those of her own age in that building? were they subject to inflammatory diseases? and what course did croup follow in this child? It is certain that the epidemic, from accumulating the sick, is different under different circumstances. Though dysentery is so frequent in camps, fleets, and perhaps military hospitals, yet in the hospitals of the metropolis, it is rarely, if ever, the form which an epidemic fever assumes. In common goals, dysentery I believe rarely occurs. In Newgate it has not been known as an epidemic, in the memory of any person of whom I have made inquiries, though prisoners have sometimes been received with that disease upon them. This is enough to show that, if croup did not spread in a workhouse, we are not from thence to determine that it is never contagious. The fact is, however, important, and the mention of it makes one wish to know the progress of such a disease in such a dwelling.

This leads me to offer a few remarks on the treatment of croup. These might, perhaps, be very well spared, after the number of respectable names cited above: but a medical paper hardly appears finished in any other way. From the various circumstances under which the membrane may be formed, it cannot be necessary to add that the mode of cure must be different; yet, when croup appears with its formidable

---

* Memoirs of Lon. Med. Society, vol. v. p. 176.

† Transactions of Society for the Improvement of Lon. Med. and Chirur. Knowledge, vol. ii. p. 25.

symptoms, or even when we have sufficient reason to apprehend it, there can be only one opinion respecting its early treatment. Nor should we for the most part be restrained in the use of the lancet, by any previous knowledge of the patient's condition. That condition, if not plethoric, must be either the usual standard of health, or delicate, or convalescent from some previous disease. Whichever it may be, such an inflammation must be arrested. It has been with much accuracy remarked, by many writers above referred to, that their patients have appeared in the eve, if I may so say, of the disease, in better spirits than usual. There cannot be a stronger indication that such appearance was only the beginning of an increased action in all the functions of life, which, if continued beyond a certain degree, must produce disease. The only situation in which we can have any question as to free evacuation, is where the diet of the patient has been habitually less than is consistent with the common standard of health. In constitutions much reduced by long privations, we sometimes find feeble attempts at inflammation, they soon end in slough, and certainly will not bear very free evacuations. These are the only cases in which we should be doubtful, and in these the symptoms will very soon direct us.

But it must be recollected, that, even in other subjects, the subduing of inflammation is not our only indication. We have not only inflammation, but that process carried on in a manner different from its usual progress, in parts so constructed and destructive to the functions of the organ on which it is seated. We must also recollect that under certain circumstances very high inflammation is not necessary to produce such an effect. It is therefore absolutely necessary to alter the action as well as to subdue its violence. In an animal body there are two means by which an action may be altered. The first is by inducing increased action in a different part; the second, by exciting a new action in the whole system. Emetics I conceive have proved successful by the first mode. It is worthy of remark, so entirely free from inflammation is the æsophagus and stomach, in croup, that the patient rarely feels any other pain from swallowing, that what arises from its interruption to breathing. An action therefore excited in the stomach and æsophagus, may prove a relief to the trachea. At the same time it remains for the practitioner to calculate what danger may arise from the complicated functions of two such important organs. Hitherto, however, no inconvenience from emetics has been described by any author, and many instances have been related of their efficacy.

The

The other mode of suppressing such an action, is by exciting a new action in the whole system. For this purpose we have no remedy so much under our command as mercury. As the object is to produce a temporary effect as early as possible, the mercurial salts seem preferable to the slower though more certain efficacy of the crude mineral. The most minute histories of successful cases seem divided between emetics and calomel, of which the last has greatly the advantage in number. Mr. Field succeeded by each mode of treatment. Mr. Rumsey succeeded in several instances with calomel, though he is too cautious to venture a decided opinion on that remedy. Many of the American physicians speak highly of the virtues of calomel, and some of them have considered it, with emetics, as sufficient without previous bleeding. Dr. Stearns, the advocate for this mode of treatment, gives calomel in doses, which nothing but his success would seem to justify; but it should always be recollected that, where the symptoms are violent, we have no prospect of a favorable issue, unless we can induce an immediate or speedy change of action; that it will be difficult to do this in proportion to the force of the previous action, and the length of time that it has continued to increase; or, in other words, that the effect of our remedy on the constitution will be less in proportion to the violence with which the system has been occupied with the former diseased action. Hence, in the more violent forms of croup and other acute diseases, the safety, advantage, and comparatively slight effect, of calomel, in doses which might prove dangerous, if not fatal, to children in health.

Long as this paper is become, I connot conclude it without taking this opportunity of publishing a record which came into my hands by the politeness of the executor of a late celebrated physician. It is extracted from the case book of his uncle, who for full thirty years was the principal physician in the city of London. Though it has no date, many circumstances prove that the case must have occurred about the year 1755, which is about ten years before Dr. Home's publication gave us a name for this formidable disease.

"Mrs. ———, aged about 40, had a favorite child, who died of a sore throat, with whom she was almost constantly upon the bed, and kissing the child. A day or two after the child's death, viz. June the 27th, complained of a sore throat, and upon examination the tonsils were inflamed, (was blooded, and the 28th cupped;) which went off suddenly the 29th; but she still complained of pain and difficulty in swallowing. July the 1st, I saw her, when she had but little fever, was very low spirited, and crying for the loss of her child. The fauces very little affected, but she complained of some difficulty difficulty in swallowing, which I imagined was a good deal hysterical. A blister was not complied with, as they used to give a violent strangury. 3d. The swallowing worse, and the breath; all other symptoms as before. 4th. The swallowing and breathing much easier, and now spit up a great deal of phlegm, and something like matter, and a bit or two of a thing like skin; now a cough, which was very troublesome; every time she drank any thing the cough was bad; this day she said, if I could cure the cough, she would be well. 5th. The same appearances, only this evening the breath worse. 6th. A very bad night; like to be suffocated, and about two in the morning brought up a slough, 6 or 8 inches long, which was hollowed in the form of the trachea; breath very bad all this day; extremely ill in the night; died 7th, in the afternoon."

This relic would have been much more valuable had the relator witnessed the whole, and prepared his paper for publication. It does not appear that he saw the child at all; nor the mother till the fourth day of the disease. If the case was croup, it would seem to favor its contagious property; and also would produce another instance of the occurrence of such a disease in mature life, and probably in reduced health. I am, Gentlemen,

Your's, &c.

JOSEPH ADAMS.

---

*To the Editors of the Medical and Physical Journal.*

GENTLEMEN,

